# Electrical Resistance Performance of Cable Accessory Interface Considering Thermal Effects

**DOI:** 10.3390/ma16114122

**Published:** 2023-06-01

**Authors:** Kai Wu, Tianfeng Zhang, Wenxin Lin, Shaolei Wu, Yu Feng, Xiangyu Zuo, Yunxiao Zhang

**Affiliations:** 1 Electric Power Research Institute, Anhui Electric Power Company of State Grid, Hefei 230601, China; 2 College of Electrical Engineering and Automation, Fuzhou University, Fuzhou 350108, China

**Keywords:** cable accessory, silicone rubber, cross-linked polyethylene, interface, electrical properties

## Abstract

Power cables are widely used in various fields of power transmission, and cable accessories are the weakest link in power cable systems due to their complex structure and multi-layer insulation coordination issues. This paper investigates the changes in electrical properties of the silicone rubber/cross-linked polyethylene (SiR/XLPE) interface at high temperatures. The physicochemical properties of XLPE material under thermal effects with different times are characterized through FTIR, DSC, and SEM tests. Finally, the mechanism of the effects of the interface state on the electrical properties of the SiR/XLPE interface is analyzed. It is found that with the increase in temperature, the changes in electrical performance of the interface do not show a monotonic downward trend, while interestingly, they can be divided into three stages. Under the thermal effects for 40 d, the internal recrystallization of XLPE in the early stage improves the electrical properties of the interface. In the later stage of thermal effects, the amorphous region inside the material is severely damaged and the molecular chains are severely broken, resulting in a decrease in the electrical properties of the interface. The results above provide a theoretical basis for the interface design of cable accessories at high temperatures.

## 1. Introduction

With the development of polymeric materials, polymer-insulated cables have been widely used in the transmission of electric energy [1]. Compared to traditional cables such as oil-filled cables and oil paper cables, cross-linked polyethylene (XLPE) cables are widely used in transmission lines due to their environmental cleanliness and excellent insulation performance [2,3]. The cable transmission system mainly consists of the cable body, cable accessories, and terminal joints. At present, with the rise of long-distance ultra-high voltage transmission technology, cable accessories are widely used for the transmission of electric energy [4]. After decades of development, the insulation problem of the cable body has been improved, while the problem of cable joints remains prominent [5,6]. The common cable accessories are usually composed of a variety of different solid media, thus forming a variety of different interfaces, mainly including the interfaces of cable main insulation—semi-conductive layer, cable main insulation—accessory insulation, stress cone—accessory main insulation, and so on. The interface composed of the main insulation of the cable and the insulation of the accessories is the most critical, and the subsequent “cable joint interface” in this article also refers to this interface.

Due to the existence of the cable joint interface, cable accessories have long been the weakest link in cable systems [7,8,9,10]. According to the analysis of AC cable failure cases, about 80% of cable failures are caused by cable accessories. Therefore, many scholars have conducted research on the failure of cable accessory interfaces.

The interfacial breakdown between two different insulation materials is completely different from bulk breakdown and surface flashover phenomena [5]. The breakdown of the interface is not only related to the insulating properties of the dielectrics but also related to the contact state of the interface and the filler of the interfacial micro gap [6]. C. Dang et al. [11,12] studied the AC breakdown characteristics of the interface between two-layer dielectrics, finding that the interfacial breakdown voltage increases with the increase in the interfacial pressure and that aging can reduce the interfacial pressure, thereby reducing the interfacial breakdown voltage. E. Kantar et al. [6,13,14] conducted some research on the effects of interfacial roughness, interfacial pressure, material elastic modulus, and composite media fillers on the interfacial breakdown strength of double-layered media. It was found that the AC breakdown voltage of the interface increases with the increase in the interfacial pressure, and the smaller the elastic modulus, the more significant the impact on the interface pressure. B. Du et al. [15,16] studied the changes in the breakdown voltage between the interface of a two-layer medium with factors such as roughness and interfacial pressure. They found that the greater the roughness of the interface, the stronger the discharge light at the interface, and the wider the damage channel of the electrical trace. In addition, with the increasing interfacial pressure, the fractal dimensions of the carbonization decrease.

The normal operating temperature of the cable is generally 90 °C, and the temperature can even reach 150 °C when there is a short circuit or other faults in the cable [17]. Therefore, cable insulation will suffer from long-term thermal effects. Under high temperatures, the degradation of XLPE material drops sharply in insulation performance. H. Li et al. [18] found that at 100 °C, thermal aging mainly affects the amorphous region; however, under thermal aging at 160 °C, the insulation is in a molten state, resulting in a transition from the crystalline region to the amorphous region of the material. Kim et al. [19] have suggested the impact of thermal aging on the insulation performance and breakdown characteristics of XLPE cables and found that thermal aging is reflected in the destruction of the molecular chain of XLPE at the micro level, while at the macro level, it is reflected in the reduction in the breakdown strength of XLPE insulation. As for silicone rubber (SiR) materials, mainly used in cable accessories, Y. Zhang et al. [20] established a physical weak zone model of SiR based on shell core results and found that electric properties, such as electrical tree characteristics, show a trend of first increasing and then decreasing. However, little attention has been paid to the electrical performance of cable accessory interfaces considering the thermal effects.

The aim of this paper is to investigate the electrical resistance performance of the interface considering thermal effects. The electrical properties (including interfacial breakdown voltage and interfacial resistivity) of the interface at different temperatures are studied. The rules of temperature on the electric properties of the interface are revealed. Moreover, thermal effects tests of XLPE samples are carried out at 100 °C for 40 days. In addition, the physicochemical properties and electric properties of XLPE materials under thermal effects are analyzed. Then, the influence mechanisms of material interface changes on the electrical properties of the interface under thermal effects are discussed. Finally, the suggestions for the operation and maintenance of cable accessories engineering are discussed and pointed out.

## 2. Materials and Methods

### 2.1. Specimen Preparation

Generally speaking, the polymeric material for cable main insulation is XLPE, and the accessory insulation is generally SiR or EPDM rubber. This article selects a double-layer dielectric interface composed of XLPE and SiR to simulate the cable accessory interface. The SiR specimen is made of two-component high-temperature vulcanized SiR (Zhonglan Chenguang, Chengdu, China), mixed evenly in a 1:1 ratio, and heated at 165 °C for 10 min at a pressure of 5 MPa under thermal vulcanization. The size of the SiR samples is 40 × 40 mm, with a thickness of 2 mm. The XLPE specimen is made of XLPE particles (Borealis AG, Vienna, Austria) vulcanized by a flat vulcanization machine, and the vulcanization conditions are as follows: pre-crosslinking for 10 min at 120 °C and 15 MPa; the cross-linked sample is obtained by crosslinking at 180 °C and 15 MPa for 8 min.

In the actual installation process of cable joints, in order to prevent semi-conductive particles from remaining on the main insulation surface, sanding paper is used to polish the main insulation surface. In this article, the surface of XLPE is uniformly polished with 1000-grit paper, and then cleaned with anhydrous ethanol. The size of the XLPE specimen is 40 × 40 mm with a thickness of 1 mm. As for the tests of thermal effects, XLPE specimens are arranged inside high-temperature ovens at temperatures of 100 °C. Specimens are taken out every 10 days for a total time of 40 days.

### 2.2. Materials Characterization

The interfacial microstructure morphology of XLPE specimens is observed by scanning electronic microscopy (SEM; Verios G4, Thermo Fisher Scientific, Waltham, MA, USA).

Infrared spectroscopy can be used to characterize the changes in functional groups of materials before and after thermal effects. A Fourier transform infrared spectrometer (Avatar 360, Madison, WI, USA) is used to test XLPE specimens at different times of thermal action, with a scanning range of 4000 cm^−1^–500 cm^−1^. Then, the carbonyl index is calculated by repeating each test 3–5 times.

Differential scanning calorimerty (DSC) is usually used to evaluate the crystallization behavior of polymers. The XLPE specimens are tested with the differential scanning calorimetry method (DSC 214 Polyma, Netzsch, Selb, Germany) in the argon atmosphere. All groups are carried out 3–5 times, and one of the typical DSC curves of each group is shown in this paper. Two heating cycles are performed. The heating temperatures range from 25 °C to 150 °C. The heating and cooling rates are both 10 °C/min. The data obtained from the second heating process are used to analyze the crystallization characteristics of the XLPE. The average crystallinity of the XLPE specimen can be expressed as follows:

(1) $$\chi =\frac{\Delta H_{m}}{\Delta H_{0}}\times 100\%$$

where $\Delta H_{m}$ is the melting enthalpy of the specimen and $\Delta H_{0}$ is the melting enthalpy with a crystallinity of 100%. Here, as for XLPE materials, $\Delta H_{0}$ = 287.3 J/g.

### 2.3. Interfacial Breakdown Experiment

**Figure 1:**
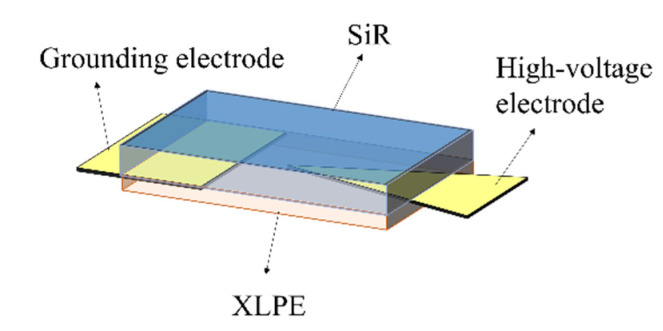
Interfacial specimen and electrode arrangement.

Due to the electric field concentration caused by the root of the stress cone root or the presence of interface defects, a needle plate electrode structure is used to simulate the related electric field concentration phenomenon, and the detailed interfacial specimen and electrode arrangement are shown in Figure 1. The interfacial electrode is copper foil with a thickness of 0.05 mm. The angle of the high-voltage electrode is 30° and the distance between the tip of the high-voltage electrode and the grounding electrode is 500.0 mm.

**Figure 2:**
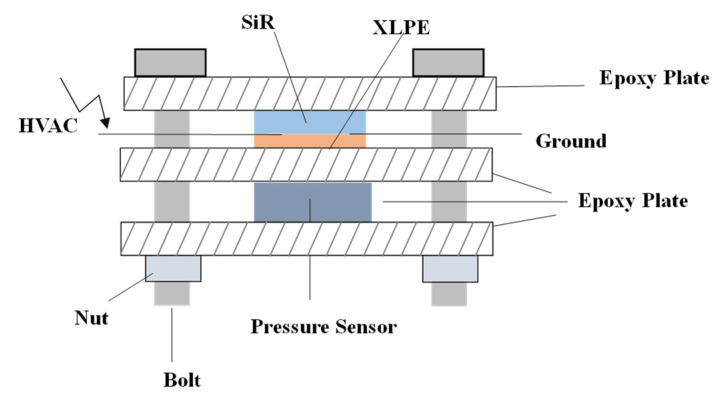
Double-layer dielectric interfacial pressure device.

The application device of interfacial pressure is shown in Figure 2, which mainly consists of three organic glass plates, bolts, nuts made of epoxy resin material, and pressure sensors. The aperture of the four corners of the organic glass plate is slightly larger than the diameter of the bolt, so the force between the bolt and the glass plate can be ignored. The pressure sensor is placed between the upper two plates. The SiR/XLPE specimen (as shown in Figure 1) is placed between the lower two plates. Before the measurement, the pressure is transmitted between the epoxy plates through four bolts by means of rotating the nuts and the indication of the pressure sensor can be considered as the force exerted on the sample. A pressure of 0.1 MPa of the interface is chosen in all interfacial breakdown experiments.

As for the study of temperature effects, the testing temperature of 25–100 °C is controlled by an air-drying oven. Before the experiment, the double-layer dielectric specimens and interfacial pressure device are placed into the oven for 30 min. When considering the effects of thermal action for a long time, a fixed temperature of 25 °C is chosen. During the AC breakdown tests, the AC voltage is increased at a constant rate of 0.5 kV/s until interfacial breakdown occurs. Due to the significant uncertainty of the breakdown of polymer, in order to better analyze the results, each group of the breakdown tests is repeated 10 times, and the average and statistical variance of these 10 groups of results is calculated as the breakdown voltage of the SiR/XLPE interface. 

### 2.4. Interfacial Resistivity Experiment

**Figure 3:**
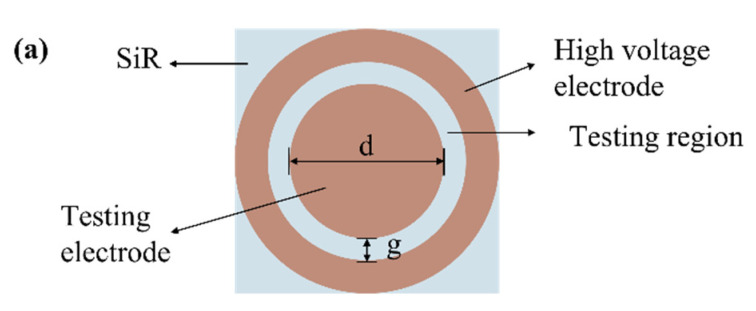

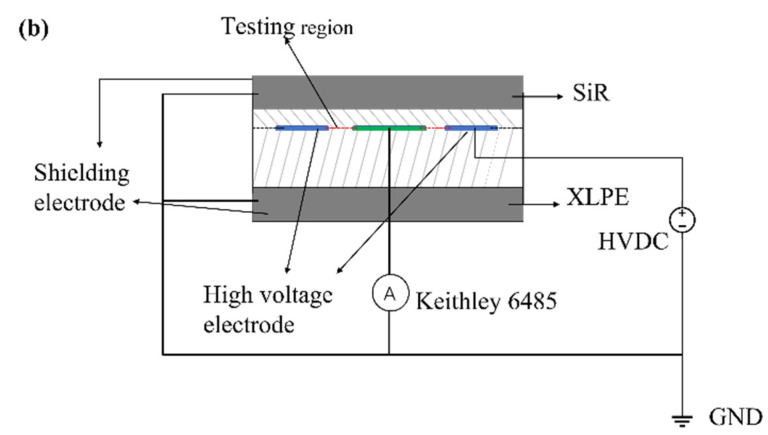
Interfacial resistivity measurement: (**a**) interfacial electrode arrangement; (**b**) schematic diagram of interfacial resistivity testing.

The interfacial resistivity of the double-layer dielectric is measured using a four-electrode system [21,22], an interfacial pressure device, and the Keithley 6485. The arrangement of the interfacial electrodes and the wiring diagram of the testing circuit is shown in Figure 3. The four-electrode system includes two interfacial electrodes in SiR/XLPE and shielding electrodes on the upper and lower surfaces of the double-layer dielectric. Copper foil tape with a thickness of 0.05 mm is adopted for all electrodes.

As shown in Figure 3a, the diameter (*d*) of the test electrode is 24 mm, and the gap (*g*) between the high-voltage electrode and the test electrode is 2 mm. Before the experiment, the four-electrode system is placed into the interfacial pressure device. The interfacial pressure is set to 0.1 MPa by tightening the nuts. The temperature regulation is the same as shown in Section 2.3. The external DC high-voltage source which is connected to the high-voltage electrode generates a high voltage of 1 kV. The Keithley 6485 connected to the testing electrode collects the current flowing through the testing sample interface. The samples are polarized for 10 min.

The average value of the current in the last 10 s of the total measurement time is taken as the steady-state current *I_sc_*. According to IEC standards, the calculation equation for the interfacial resistivity (*ρ*) of double-layer media can be calculated by Equation (2). A total of 3–5 experiments per group are conducted and the interface resistivity of the relevant group is calculated by averaging their values.

(2) $$\rho =\frac{U}{I_{sc}}\times \frac{\pi (d+g)}{g}$$

## 3. Electrical Properties of the Interface at Different Temperatures

### 3.1. Interfacial Breakdown Voltage

**Figure 4:**
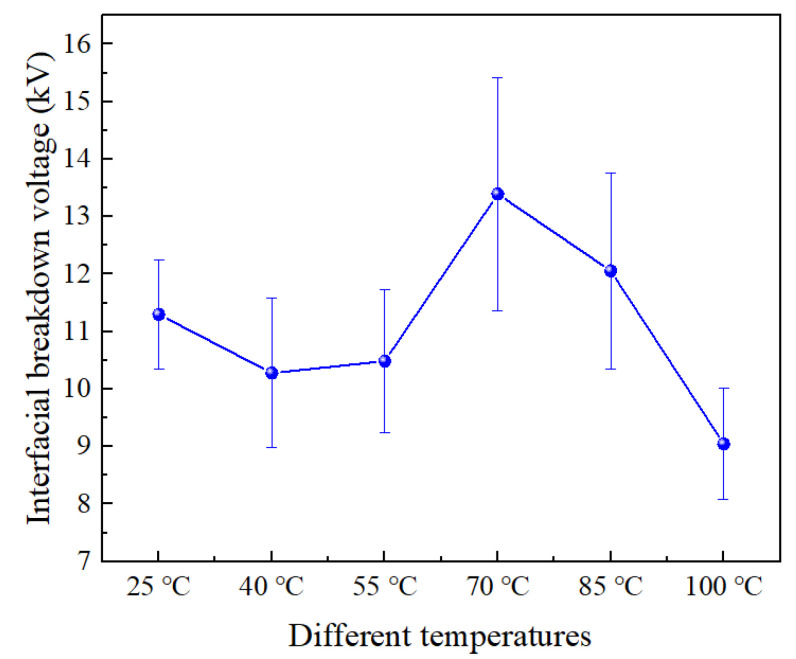
Interfacial breakdown voltage at different temperatures.

Figure 4 shows the interfacial breakdown voltage at different temperatures. At 25 °C, the interfacial breakdown voltage of SiR/XLPE is 11.3 kV. When the temperature rises to 50 °C, it remains basically unchanged. However, with the increase in the temperature to 70 °C, the breakdown voltage reaches the maximum value (13.4 kV), increasing by 18.6% compared to that at 25 °C. Interestingly, when the temperature further increases from 70 °C to 100 °C, the interfacial breakdown voltage of SiR/XLPE decreases to 9.0 kV, declining by 20.4% compared to that at 25 °C. Therefore, it can be summarized that the interfacial breakdown voltage first remains unchanged in the lower temperature and then increases with the increase in the temperature. However, at elevated temperatures, the interfacial breakdown voltage decreases with the increasing temperature.

### 3.2. Interfacial Resistivity

**Figure 5:**
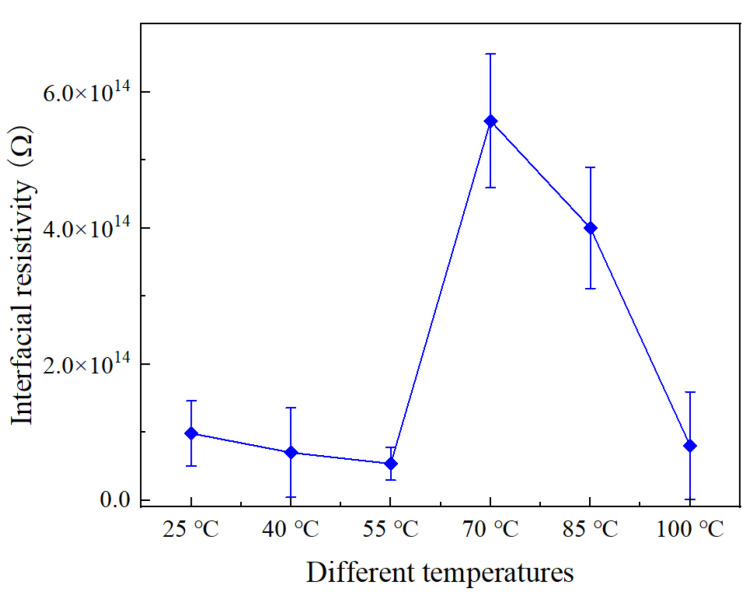
Interfacial resistivity of SiR/XLPE under different temperatures.

Figure 5 shows the interfacial resistivity of SiR/XLPE at different temperatures, while its trend is similar to the trend of the interfacial breakdown voltage. It is found that the interfacial resistivity of SiR/XLPE is 9.8 × 10^13^ Ω at 25 °C. When the temperature rises to 55 °C, it decreases to 5.4 × 10^13^ Ω, a decrease of 44.9% compared to that at 25 °C. Moreover, it reaches 5.8 × 10^14^ Ω at 70 °C, which is the maximum among all specimens. With the further increase in temperature from 70 °C to 100 °C, the interfacial resistivity decreases by 86.2% compared to that at 70 °C.

### 3.3. Effects of Temperature on Electrical Properties

The conductivity of polymers is greatly influenced by the temperature and electric field. Hjerrod et al. analyzed the trend of conductivity changes in materials at different temperatures. The material conductivity function of the temperature and electric field could be shown as follows [23]:

(3) $$\gamma =A\exp(\frac{-\varphi q}{k_{b}T})\frac{\sinh(B\;|\dot{E}|)}{\left|\dot{E}\right|}$$

where A is a constant related to the material; B is a constant related to the electric field; *φ* is the activation energy of the material; *q* is the charge amount of the carrier; and *k_b_* is the Boltzmann constant.

**Figure 6:**
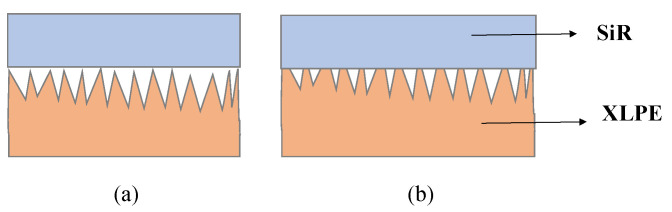
Schematic diagram of the contact state of the interface: (**a**) at low temperature; (**b**) at high temperature.

From Equation (3), the conductivity of both XLPE and SiR materials increases with the increase in temperature. However, the electrical properties of the double-layer interface are related to not only the insulation properties of XLPE and SiR but also the contact state, interface fillers, etc. Figure 6 shows a schematic diagram of the contact state between SiR and XLPE. The SiR is equivalent to a smooth medium in the contact diagram due to the fact that the surface of the SiR has not been polished with sandpaper. Usually, the melting point of XLPE is within the range of 85–110 °C. As the temperature increases, the temperature of XLPE material gradually approaches its melting point and becomes softer. Therefore, under a certain pressure, the interface gap of the double-layer medium decreases with the increased contact area between the XLPE and SiR, as shown in Figure 6b. Usually, the dielectric constant of gas is less than that of the solid dielectric; the dielectric breakdown or partial discharge of gas occurs more easily at a lower voltage. Thus, with the increase in temperature, the interface gap decreases, and the interface breakdown voltage increases.

When the temperature increases further, although material softening at high temperatures can cause a decrease in interfacial voids, charge carriers are injected into the bilayer dielectric interface through the high-voltage electrode in a thermal ionization manner according to Richardson Schottky’s law. In addition, according to the theory of solid media, the energy band of the medium tilts under the action of an external electric field, so the carriers captured by the interface traps of SiR and XLPE obtain sufficient energy, which makes it easier to cross the material’s trap barrier and reach the interface. Therefore, the number of charge carriers at the interface of the double-layer dielectric increases sharply, indicating a significant downward trend in the interfacial breakdown voltage.

Normally, interfacial resistivity can be used to evaluate the difficulty of charge transfer or current flow along the interface. The lower interfacial resistivity indicates the leakage current can more easily flow along the interface. The bulk conductivity of XLPE and SiR increases with the increase in temperature, as shown in Equation (3), which also makes the current flow more easily along the interface of the double-layer medium, resulting in a decrease in interfacial resistivity. The XLPE becomes softer when the temperature of XLPE gradually approaches the melting point, and the contact area of the double-layer dielectric interface increases, which makes the interface gap decrease, resulting in a decrease in the number of charge carriers at the interface. Thus, the interfacial resistivity will be improved. When the temperature gets higher, similar to the effects on the breakdown voltage, the number of charge carriers increases sharply at elevated temperatures. With the increase in leakage current, the interfacial resistivity decreases.

## 4. Electrical Properties of Interface under Thermal Effects with Different Time

### 4.1. FTIR Spectroscopy

**Figure 7:**
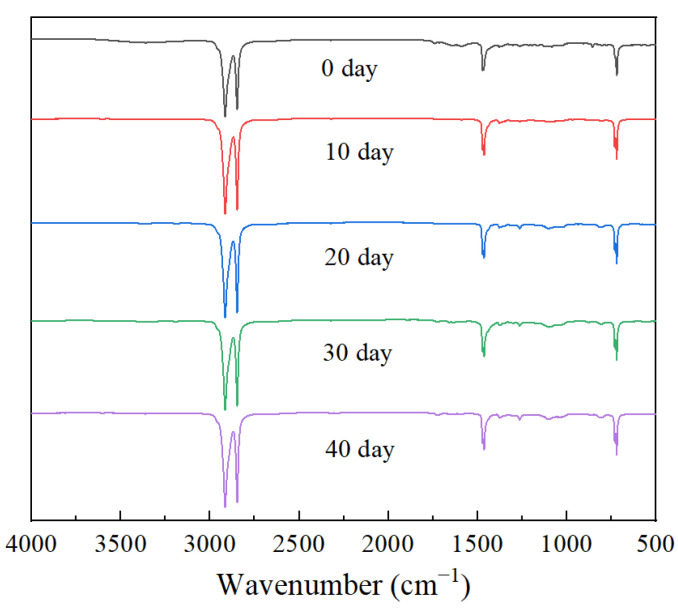
Infrared spectra of XLPE specimen under different stages of thermal action.

The position and intensity of characteristic peaks in infrared spectroscopy can effectively characterize the state of materials. Figure 7 shows the Fourier transform infrared spectra at different times. For unaged XLPE, the peaks of 2914 cm^−1^ and 2848 cm^−1^ correspond to the asymmetric and symmetric stretching of methylene (-CH_2_), respectively. The peak of 1470 cm^−1^ is the bending vibration of methylene (-CH_2_) and the peak of 721 cm^−1^ is the rocking vibration of methylene (-CH_2_) [24].

As shown in Figure 7, there is no significant change in the infrared spectrum of XLPE after 40 d of thermal effects, especially in the wavenumber range of 1730 cm^−1^–1725 cm^−1^. The wavenumber range corresponds to the carbonyl (-C=O) absorption peak, which can be used as a characteristic peak to characterize the thermal oxygen reaction. It is indicated that XLPE did not occur or experience a slight oxidation reaction during this process. It is worth noting that after 40 d, the infrared spectrum of XLPE exhibits some weak absorption peaks in the wavenumber range from 1300 cm^−1^ to 1000 cm^−1^. According to the literature, these absorption peaks are related to the vibration of the ether group (-C-O-C) [25,26].

**Figure 8:**
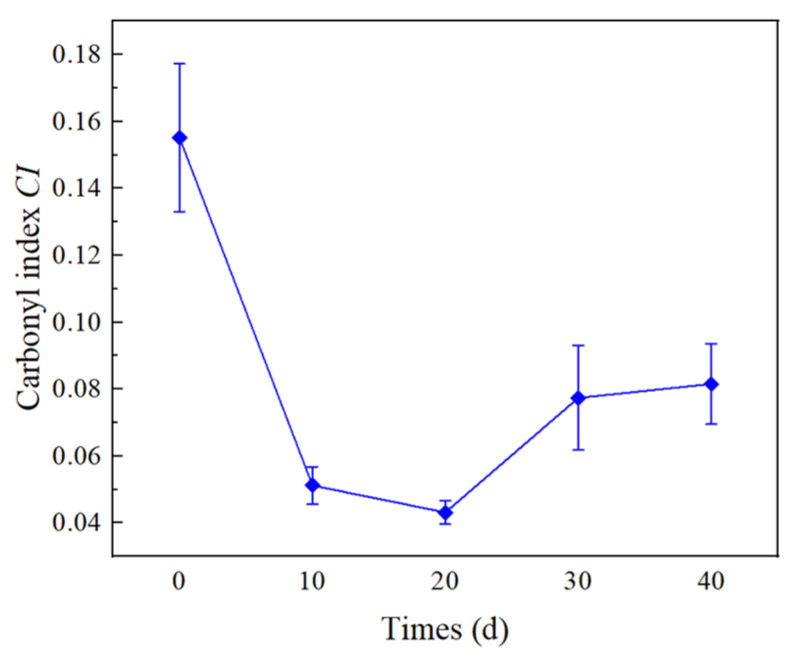
Carbonyl index of XLPE specimen at different stages of thermal action.

In order to further evaluate the changes in carbonyl groups in XLPE during the thermal process, the carbonyl index *CI* is used for quantitative characterization, which can be expressed as follows [24]:

(4) $$CI=\frac{A_{1732}}{A_{1466}}$$

where *A*_1732_ and *A*_1466_ represent the absorption strengths at 1732 cm^−1^ and 1466 cm^−1^, respectively. Figure 8 shows the trend of *CI* of XLPE specimens at different times. The *CI* of the unaged XLPE material is relatively high, which may be related to additives such as antioxidants in the material. During the early thermal process (0–20 d), the *CI* shows a decreasing trend as antioxidants are gradually consumed. After further thermal effects, when the antioxidant in XLPE is completely depleted, the XLPE specimen begins to enter the oxidation reaction. With the formation of polar groups, the carbonyl index of XLPE increases.

### 4.2. DSC Results

**Figure 9:**
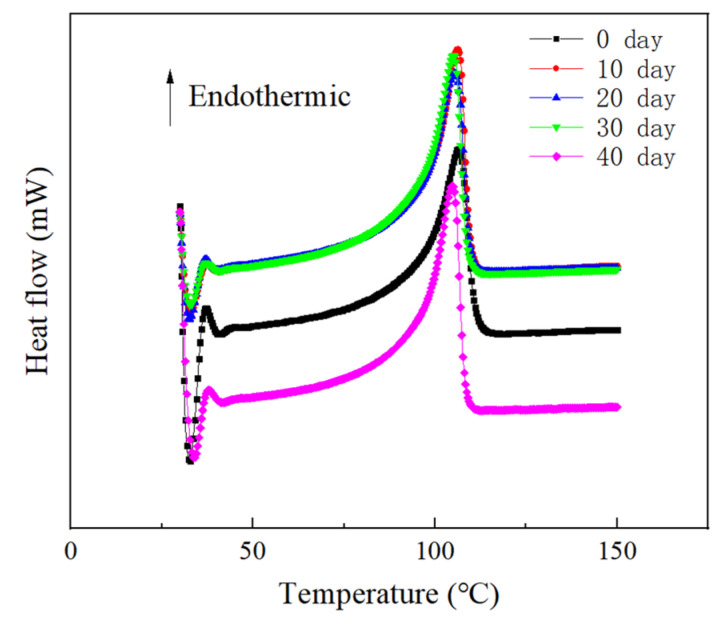
DSC curves of XLPE specimens on different days.

As a semi-crystalline polymer, the properties of XLPE are quite dependent on the aggregated structure. Therefore, the melting and crystallization behavior of XLPE obtained from the DSC curves is of great significance for analyzing the physical and chemical properties of XLPE. Due to our testing of 3–5 sets of DSC results, Figure 9 just shows typical DSC curves of the XLPE at different stages of thermal action. It can be seen from Figure 9 that there is no significant change in the temperature corresponding to the melting peak of the XLPE sample, as well as in the strength and width of the melting peak. Corresponding to Figure 9, the crystallinity and melting temperature of the XLPE are shown in Table 1.

From Table 1, the crystallinity (*χ*) of unaged XLPE is 31.7%. The *χ* of XLPE reaches its maximum value for 10 d. With the deepening of thermal action, the *χ* of XLPE does not show significant changes. According to the results of crystallization, the crystalline structure of XLPE is not significantly damaged during the thermal action process. In addition, the melting temperature of XLPE shows a trend of first increasing and then decreasing. The highest melting temperature of XLPE is 106.4 °C for 10 d.

### 4.3. SEM Images

**Figure 10:**
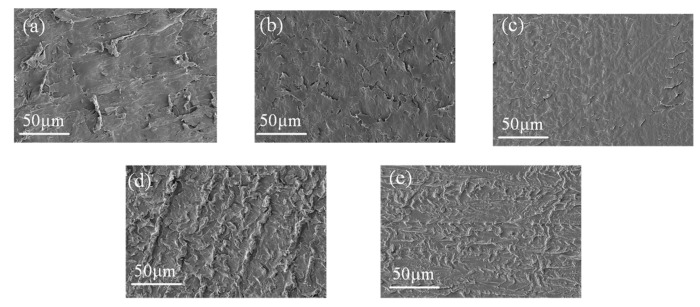
SEM images of XLPE at different times of thermal action: (**a**–**e**) 0, 10, 20, 30, 40 d, respectively.

Figure 10 shows the SEM images of XLPE samples on different action days. The unaged XLPE surface is polished with sandpaper and the surface chips are slightly rolled. The surface of XLPE gradually becomes smoother after 20 d. However, as shown in Figure 10d,e, with further thermal action, the surface of XLPE can be observed as obviously having an aggregated structure.

### 4.4. Interfacial Breakdown Voltage and Resistivity of SiR/XLPE 

**Figure 11:**
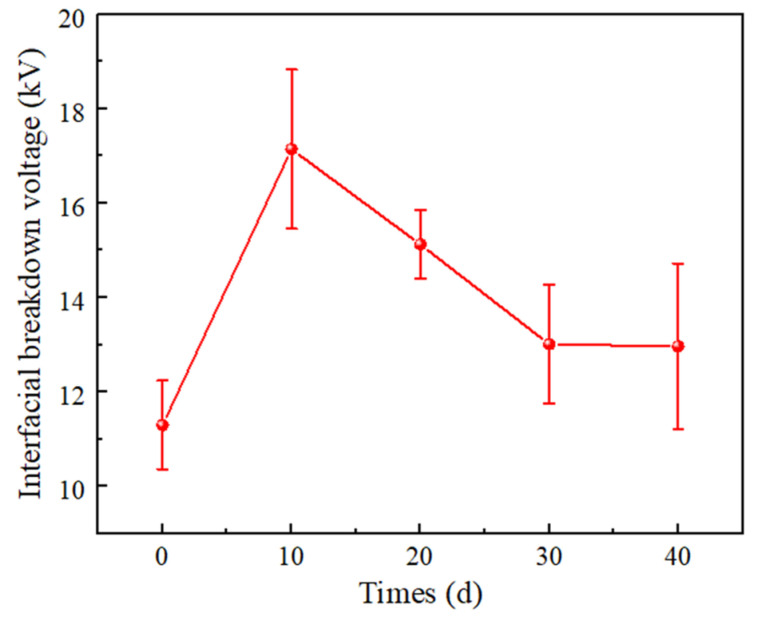
Interfacial breakdown voltage of SiR/XLPE at different times of thermal action.

Figure 11 shows the breakdown voltage of the double-layer dielectric interface at different times of thermal action. When XLPE is not aged, the breakdown voltage at the SiR/XLPE interface is 11.3 kV. After 10 d, the breakdown voltage of the interface increases to 17.1 kV, increasing by 51.3%. With the increase in the time from 10 d to 30 d, the breakdown voltage is reduced to 13.0 kV, decreasing by 24.0% compared to that of 10 d. It is worth noting that after 40 d, the interfacial breakdown voltage is almost the same as for 30 d.

**Figure 12:**
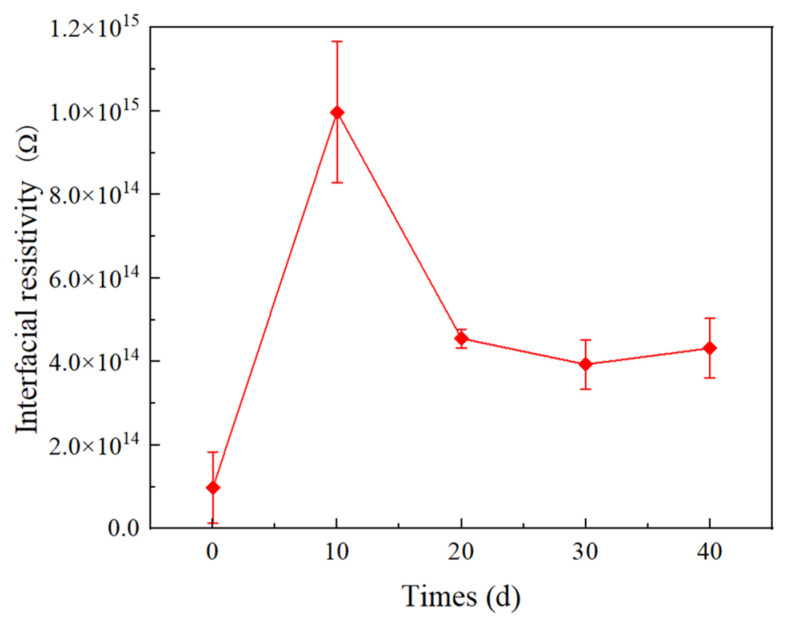
Interfacial resistivity of SiR/XLPE at different times of thermal action.

Figure 12 shows the interfacial resistivity of double-layer media at different times. For unaged XLPE, the interfacial resistivity of SiR/XLPE is 9.8 × 10^13^ Ω. When the time reaches 10 d, the interfacial resistivity of the double-layer dielectric reaches the maximum value, which is increased by 90.2% compared to the unaged specimen. When it increases from 10 d to 30 d, the interfacial resistivity decreases to 3.9 × 10^14^ Ω and the interfacial resistivity is almost the same as that of 30 d.

### 4.5. Discussions

#### 4.5.1. The Physicochemical Properties of XLPE under Thermal Effects

**Figure 13:**
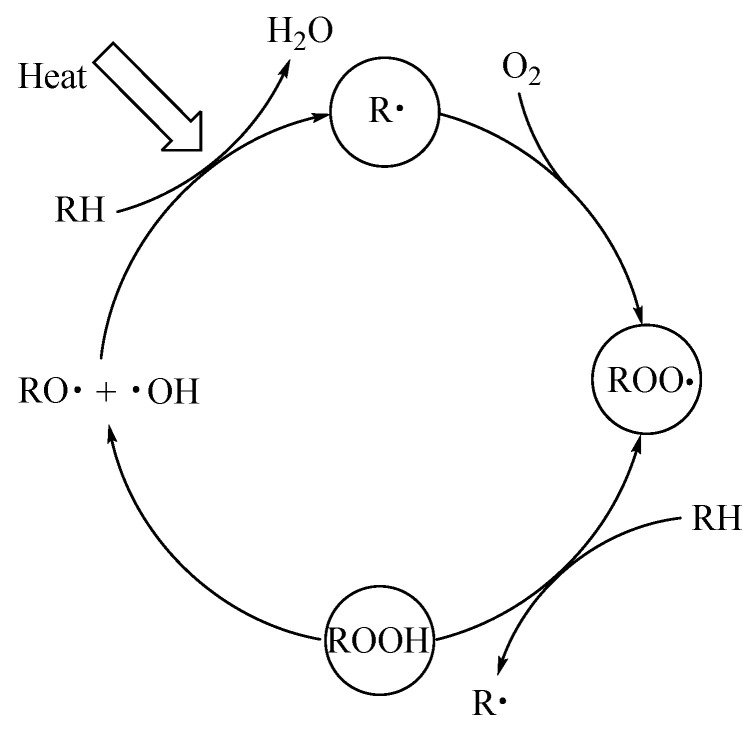
Schematic diagram of the thermal oxygen reaction of XLPE.

It is well known that XLPE is a semi-crystalline material composed of crystalline and amorphous regions [27]. The crystalline region of XLPE exists in the form of spherical crystals, which are mainly composed of radially arranged and ordered lamellar ribbons [28]. During the thermal action process, the branched chains of XLPE are first cut off. In the presence of oxygen, the reaction of radicals (R·) formed in the initial chain scission step with available oxygen leads to the formation of hydroperoxides radicals (COO·), which may react with XLPE, resulting in further breakage of XLPE chains. The specific thermal oxygen reaction of XLPE is shown in Figure 13.

According to the changes in the surface aggregation structure of XLPE, as shown in Figure 8, Figure 9 and Figure 10, the changes in the interfacial physicochemical properties of XLPE can be divided into two stages. The first stage (0–20 d) is the recrystallization stage, where the oxidation reaction is inhibited due to the presence of antioxidants. Therefore, the amorphous region of XLPE is mainly affected during this process. In addition, the mobility of molecules formed by molecular chain breakage is enhanced, and recrystallization occurs due to the mutual contact of small molecules, leading to the formation of large molecular chains.

The second stage (20–40 d) is the oxidation reaction stage, the antioxidants inside the material are consumed and the oxidation reaction can proceed spontaneously. The polar molecular chains formed during the oxidation reaction are more likely to accumulate, which makes XLPE exhibit a clear aggregation state. In addition, the crystalline structure of XLPE is not significantly damaged during this process.

#### 4.5.2. Influence of Thermal Effects on Electrical Properties of the Double-Layer Dielectric Interface

The dense arrangement of macromolecular chains in the crystalline region of XLPE material is not conducive to the migration of charge carriers; however, the molecular chains in the amorphous region of XLPE are loosely arranged, making it easier for charge carriers to move within the amorphous region.

In the initial stage of thermal action, the crystallinity of XLPE increases due to the recrystallization, and the arrangement of macromolecular chains becomes more regular, which makes it difficult for carriers to flow along the interface and for the formation of breakdown channels. Meanwhile, as shown in Figure 10, in the early stage of thermal action, the recrystallization of XLPE makes the XLPE smoother, which leads to smaller air gaps at the interface formed by XLPE and SiR under certain pressure. Thus, the breakdown voltage at the interface increases with time in the earlier thermal action process. In addition, the number of charge carriers decreases, leading to a decrease in leakage current along the interface, which results in an increase in interfacial resistivity.

In the later thermal process, the XLPE molecular chains are severely broken. Factors such as carriers trap or ionization centers, which are served by low-weight molecule products, affect the generation and the transport of carriers [29,30]. The increase in time leads to an increase in small molecule products. Moreover, as the time increases, the surface roughness of XLPE increases, as shown in Figure 10. Thus, the interface electrical properties (including interfacial breakdown voltage and resistivity) decrease with increasing time in the later stages of the thermal action process.

## 5. Conclusions

This paper concerns the breakdown voltage and resistivity of the interface of SiR/XLPE considering thermal effects. Subsequently, the physicochemical properties of XLPE samples are characterized. The specific conclusions are as follows:

As for the effects of temperature on the electrical properties of the interface, as the temperature increases, the interface electrical performance of SiR/XLPE can be divided into three stages. At lower temperatures, the electrical properties of the interface slightly decrease due to the increase in electrical conductivity with increasing temperature. Later, as the XLPE material gradually approaches its melting point, the contact area of the SiR/XLPE interface increases and the interface gap decreases, resulting in an increase in the electrical performance of the double-layer dielectric interface; however, with the further increase in temperature, the number of carriers flowing through the interface increases, and the interface breakdown voltage and interface resistivity both show a downward trend. The influence of temperature on the electrical performance of the interface could provide a theoretical basis for the insulation structure and threshold design of cable accessories.

In the thermal action process, the physicochemical properties of XLPE can be divided into two stages. The earlier stage is the recrystallization process. The oxidation reaction could be restrained by the antioxidant. During the recrystallization stage, the crystallinity of XLPE increases significantly, where the macromolecular chains of XLPE are arranged more neatly. Thus, the electrical properties of the SiR/XLPE interface increase with the increasing time in this stage. The second stage is the oxidation reaction stage since the antioxidant has been completely consumed. The amorphous region within the material is severely damaged. The increase in time leads to an increase in small molecule products and an increase in the surface roughness of XLPE. Thus, the interfacial electrical properties decrease with the increasing time of thermal action. The results of long-term thermal action on the electric performance of the interface remind us to pay more attention to the changes in the cable interface under long-term thermal action, which is crucial for cable maintenance and condition assessment.

## Figures and Tables

**Table 1:** The crystallinity and melting temperature of XLPE.

Thermal Action Time/d	χ/%	*T*_m_/°C
0	31.7	106.2
10	34.3	106.4
20	32.7	105.4
30	33.3	105.1
40	33.9	104.7

## Data Availability

Not applicable.
